# Aged black garlic extract inhibits the growth of estrogen receptor-positive breast cancer cells by downregulating MCL-1 expression through the ROS-JNK pathway

**DOI:** 10.1371/journal.pone.0286454

**Published:** 2023-06-23

**Authors:** Qiwei Yang, Fang Li, Guohui Jia, Rui Liu

**Affiliations:** 1 Inner Mongolia Medical University Third Clinical Medical College, Inner Mongolia Autonomous Region, Baotou, China; 2 Department of Experimental Center, The Third Affiliated Hospital of Inner Mongolia Medical University, Inner Mongolia Autonomous Region, Baotou, China; 3 Department of Laboratory, The Third Affiliated Hospital of Inner Mongolia Medical University, Inner Mongolia Autonomous Region, Baotou, China; 4 Department of General Surgery, The Third Affiliated Hospital of Inner Mongolia Medical University, Inner Mongolia Autonomous Region, Baotou, China; Chung Shan Medical University, TAIWAN

## Abstract

The black garlic is produced from the raw garlic by Milliard reaction at high temperature (~60–90°C) and humidity (~70–90%). In this process, the pungent odor and gastrointestinal irritation effects of the raw garlic are reduced. At the same time, unstable compounds such as allicin are converted into stable organosulfur compounds with antioxidant activity. Previous studies have confirmed that black garlic extract has anti-tumor effects and could inhibit the proliferation of various tumor cells, including breast cancer cells MCF-7. However, the mechanisms of the anti-tumor effects remain unclear. In this study, we found that the black garlic extract could inhibit the proliferation, invasion, and metastasis of estrogen receptor-positive breast cancer cells, promote their apoptosis, and inhibit their epithelial-mesenchymal transition. Mechanistically, the black garlic extract reduced the expression of the anti-apoptotic protein MCL-1, which was achieved by modulating the ROS-JNK signaling pathway. In addition, the black garlic extract also decreased the expression of BCL-2 and increased the expression of BAX and BIM. We also found that the black garlic extract, in combination with venetoclax, a BCL-2 inhibitor, synergistically kills the estrogen receptor-positive breast cancer cells. These results suggested that black garlic extract has great therapeutic value and prospects for estrogen receptor-positive breast cancer treatment.

## Introduction

Aged black garlic (ABG) is generated from raw garlic at high temperatures and humidity and is named for its overall black color after aging. With a sweet and sour taste and a jelly-like texture, ABG lacks the pungent smell of raw garlic and has become a popular healthy food in Asian countries in recent years. Compared with raw garlic, unstable compounds such as allicin have been significantly reduced in ABG. In contrast, stable bioactive compounds such as phenols, flavonoids, S-allyl cysteine (SAC), and s-allyl mercaptocysteine (SAMC) were significantly increased. The water-soluble components of the aged black garlic extract (ABGE) were mainly SAC and SMAC, and the oil-soluble components were mainly diallyl sulfide (DAS), diallyl disulfide (DADS), diallyl trisulfide (DATS) [1]. Previous studies have shown that ABGE has dose-dependent growth inhibitory effects on various tumors. It inhibits the growth of gastric cancer cells [2], leukemia cells [3], breast cancer cells [4], colon cancer cells [5], liver cancer cells, and lung cancer cells.

Estrogen Receptor-positive (ER+) breast cancer is the most common subtype of breast cancer. Although endocrine therapy has significantly reduced the recurrence and mortality rate in this type of breast cancer, *de novo* and acquired resistance to endocrine therapy remains a major problem. The members of the BCL-2 family play an essential role in the regulation of cell apoptosis. The BCL-2 family is divided into three categories: the anti-apoptotic proteins such as BCL-2, MCL-1, and BCL-XL, which mainly inhibit cell apoptosis and promote cell survival; Apoptosis executioner proteins BAX and BAK. When activated, these proteins can form oligomers to cause the mitochondrial outer membrane permeabilization (MOMP) to induce cell apoptosis; Homology-3 (BH3) -only pro-apoptotic proteins such as BIM, BAD, BID, PUMA, and NOXA. One of the roles of these proteins is to activate apoptosis executioner proteins directly.

BCL-2 is a direct target of ER. Most primary and metastatic ER+ breast cancer cells overexpress BCL-2, which makes targeting BCL-2 possible to treat this type of breast cancer [6]. Venetoclax, a highly selective BCL-2 inhibitor, has been clinically used to treat a variety of hematological malignancies, such as chronic lymphocytic leukemia and relapsed/refractory acute myeloid leukemia. And it has been observed to prolong the overall survival of these patients. A clinical study has found that venetoclax improves the tumor response to tamoxifen in ER+ breast cancer patients [7]. However, elevated MCL-1 expression makes ER+ breast cancer cells resistant to venetoclax and weakens the therapeutic effect of this drug. Therefore, simultaneous inhibition of MCL-1 and BCL-2 may further inhibit the growth of ER+ breast cancer cells and reverse cell resistance to venetoclax.

JNK is a mitogen-activated protein kinase that phosphorylates the Thr163 and Ser121 of MCL-1, which leads to the degradation of MCL-1 [8]. JNK activation is regulated by various stimuli, including reactive oxygen species (ROS). In the present study, we found that ABGE could inhibit the proliferation, invasion, and metastasis of ER+ breast cancer cells MCF-7 and MDA-MB-361, promote apoptosis and inhibit the epithelial-mesenchymal transition (EMT). ABGE increased the ROS in ER+ breast cancer cells and activated the JNK signaling pathway, thereby reducing MCL-1 expression. In addition, ABGE reduced the expression of BCL-2, increased the expression of BAX and BIM, and promoted cell apoptosis. In addition, we also found that black garlic extract could synergistically kill ER+ breast cancer cells in combination with venetoclax.

In conclusion, ABGE showed promising prospects in treating ER+ breast cancer and can serve as an adjuvant therapeutic food.

## Material and methods

### Cell culture and ABGE preparation

MCF-7 and MDA-MB-361 cells were purchased from the American Type Culture Collection (ATCC, Manassas, VA, USA). Cells were cultured in DMEM medium (Gibco, USA) supplemented with 10% fetal bovine serum (FBS, Gibco, USA), glutamine (2 mM/L; Gibco, USA), penicillin (100 U/mL; Gibco, USA) and streptomycin (100 mg/mL; Gibco, USA). All cells were cultured at 37°C in a humidified atmosphere containing 5% CO2. ABGE powder was purchased from Shanxi Cupressaceae Biotechnology Co., LTD (Shanxi, CHN). The ABGE powder was dissolved in 0.9% normal saline to make the final concentration of the solution 100mg/ml.

### Cell proliferation assay

Cell proliferation was measured by the MTT assay, CSFE assay, and BrdU assay. Briefly, for the MTT assay, cells were plated into 96-well plates at a density of 10^5^ cells/ml and cultured for 24h. The cells were treated with 100mg/ml ABGE for 24h, 48h, and 72h, then treated with 50ul of MTT reagent (1mg/ml) for another 4h. Formazan crystals were dissolved in 150ul DMSO after the culture medium was removed. A microplate spectrophotometer was used to measure absorbance at 490 nm (Bio-Rad, USA). Treatment group value/control group value *100% = cell proliferation rate. For the CSFE assay, as instructed by the manufacturer, CSFE (5-10uM, Invitrogen, USA) kit was used to measure proliferation. The CSFE fluorescent signal of the cells was analyzed using flow cytometry. For the BrdU assay, to label proliferating cells, 10 uM BrdU was added to the culture medium for 4h. Afterward, cells were fixed with 4% PFA and stained with BrdU/DAPI immunofluorescence double staining. In three independent experiments, 10 pictures of stained cells were taken, and the percentage of BrdU-positive cells was calculated.

### Cell apoptosis assay

The apoptosis assay kit (Invitrogen, USA) was used for cell apoptosis analysis. In 24 well culture plates, cells were seeded (about 5*10^5^ cells/well) and then incubated for 24 h. The cells were then given 100mg/ml ABGE or culture media (negative control). Apoptosis was then examined by Annexin V/PI staining, followed by flow cytometry analysis of stained cells. ABGE-treated cells were harvested after 48 hours, washed twice with cold PBS, and resuspended in 1X Annexin binding buffer. 6ul FITC-conjugated Annexin V and 1ul PI were added to a fresh 1.5 ml tube containing 100 ml of binding buffer and cells. In each tube, 200 ml of 1X Annexin binding buffer was added after 15 minutes of incubation. The stained cells were analyzed by flow cytometry (BD Biosciences, USA) with flowjo software (v10.6.2, Treestar, USA).

### Transwell migration and invasion assay

Corning transwell insert chambers (8mm pore size; Corning, USA) and BD BioCoat Matrigel Invasion Chambers (BD Biosciences, USA) were used to measure cell migration and invasion ability. After incubation with 100mg/ml ABGE for 24h, the cells were harvested and resuspended in a serum-free medium. About 5*10^4^ (migration assay) or 10^5^ (invasion assay) cells were added to the chamber and incubated for another 24h. Invading or migrating cells were fixed in 20% methanol, stained with 0.1% crystal violet (Invitrogen, USA), and imaged and counted.

### Wound healing assay

Following ABGE treatment and serum starvation, cells were grown in 24-well plates to about 100% confluency. The migration ability of the cells was determined by measuring the movement of cells into a scraped area created by a sterile tip. After 24 hours, wound closure was investigated and photographed using a microscope (Olympus, JP).

### Plasmid transfection

MEK1-GFP plasmids encode constitutively active MEK1 was purchased from Addgene (#14746). Plasmids were transiently transfected into MCF-7 cells using the Lipofectamine 3000 reagent (Invitrogen, US) according to the manufacturer’s instructions. The JNK inhibitor SP600,125 (10uM) was purchased from Selleckchem (US).

### Western blot analysis

The expression of proteins was analyzed by Western blot. The cells were incubated with ABGE for 24 hours and then lysed with RIPA buffer. Protein concentrations were determined using a plate reader (Bio-Rad, USA), and equal amounts of protein were loaded. Denatured proteins (25-30ug) were electrophoresed in 4–12% or 4–20% SDS-PAGE gels, transferred to PVDF membranes, and probed with specific antibodies. The antibodies used were listed below: MCL-1 (#94296), BCL-2 (#3498), BIM (#26184), BAX (#41162), and BAK (#6947) were purchased from Cell Signaling Technology; NOXA (#PRS2473), PUMA (#PRS3043), E-cadherin (#SAB4503751), N-cadherin (#MABT530), Slug (#PRS3959), Vimentin (#V6389), β-Actin (#A3854) were purchased from Sigma-Aldrich; p-JNK (ab176662), JNK (ab126424), p-P38 (ab178867), P38 (ab170099), p-ERK (ab201015), ERK (ab32537) were purchased from Abcam. Blot bands were visualized after incubating with the secondary antibodies and the chemiluminescent substrate.

### Mitochondrial membrane potential measurements

Mitochondrial membrane potential measurements were carried out using the JC-1 mitochondrial membrane potential assay kit (ab113850, Abcam, US) according to the manufacturer’s instructions. Briefly, 10^4 cells/well were seeded into a 96-well plate and allowed to attach overnight. 100ul/well of the working JC-1 solution was added per well and incubated for 10min at 37°C in the dark. The results were observed and photographed under a fluorescence microscope. The level of mitochondrial membrane potential was shown as the ratio of red to green fluorescence.

### Oxygen consumption rate (OCR) measurements

OCR values were measured by the Seahorse XF-96 extracellular flux analyzer. MCF-7 or MDA-MB-361 cells at a density of 1 × 10^4 cells/well were grown in Seahorse XF96 cell culture microplates. Before the assay, cells were washed twice with serum-free DMEM and then incubated in serum-free DMEM supplemented with 30 mM glucose and 1 mM pyruvate for 1 h, followed by mitochondrial stress testing. The tests were performed by exposing the cells to oligomycin (1 μM), FCCP (1 μM), and rotenone/antimycin (1 μM). OCR was measured and curves were plotted after each treatment.

### Intracellular and mitochondrial ROS measurements

Flow cytometry was used to measure mitochondrial and intracellular ROS. Following ABGE treatment, we collected, washed, and resuspended cells in a serum-free medium with dyes. For mitochondrial ROS measurements, MitoSOX (Sigma-Aldrich, US) was added to the cells and incubated for 10 minutes after washing. DCFHDA (Beyotime, China) was used for intracellular ROS measurements as the fluorescent reporter.

### Statistical analysis

Statistical analysis was performed using GraphPad Prism (version 9.3, GraphPad Software, US). All data were expressed as mean ± SD. One-way analysis of variance (ANOVA), following Tukey’s multiple comparisons tests, was used to analyze the data among >3 groups. The student’s t-test (unpaired, two-tailed) was used for comparisons between the two groups. The combination index (CI value) was calculated by CompuSyn software (https://www.combosyn.com/) as instructed. P values were indicated by asterisks: *p < 0.05, **p < 0.01, ***p < 0.001. All experiments were performed in triplicate.

## Results

### ABGE inhibits the proliferation of ER+ breast cancer cells

**Fig 1:**
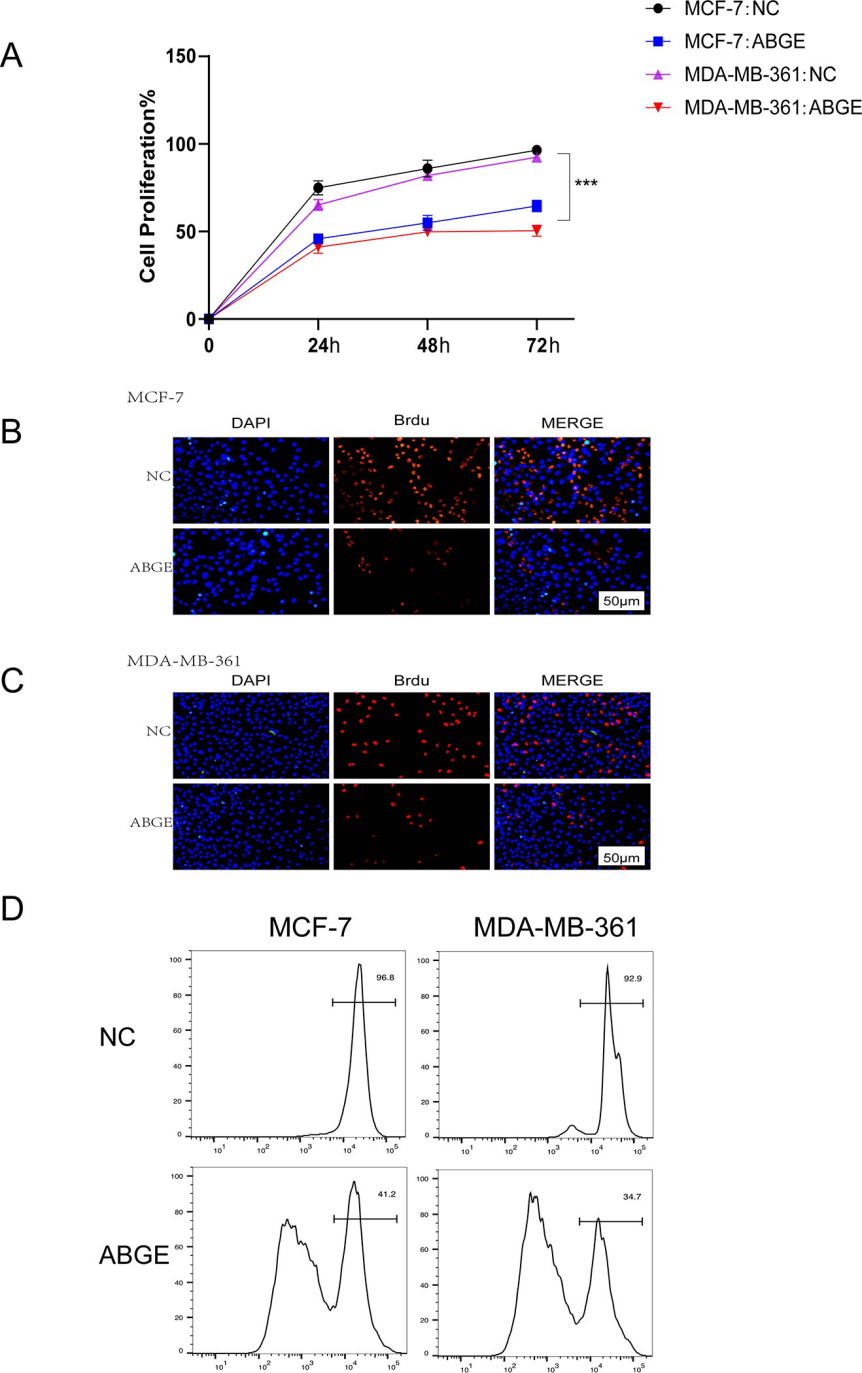
ABGE inhibit MCF-7 and MDA-MB-361 cells proliferation and promote their apoptosis in vitro. (a),Cells proliferation were detected by MTT Assay. (b,c,d),The proliferation of MCF-7 and MDA-MB-361 cells was examined by BrdU assay and CFSE assay after treatment with ABGE, respectively. *** indicates *p* value less than 0.01.

To explore the effects of ABGE on breast cancer cell proliferation, we carried out the MTT assay, CSFE assay, and BrdU assay. As shown in Fig 1A and 1B, the proliferation ability of MCF-7 and MDA-MB-361 cells was significantly reduced after being treated with 100mg/ml ABGE for 24, 48, and 72h (p<0.01). The CSFE and BrdU assay showed similar results (Fig 1B–1D). However, in normal mammary cell line MCF-10A, we did not observe an inhibitory effect of ABGE on their growth (S1 Fig).

### ABGE induces apoptosis and reduces the migration and invasion of ER+ breast cancer cells

**Fig 2:**
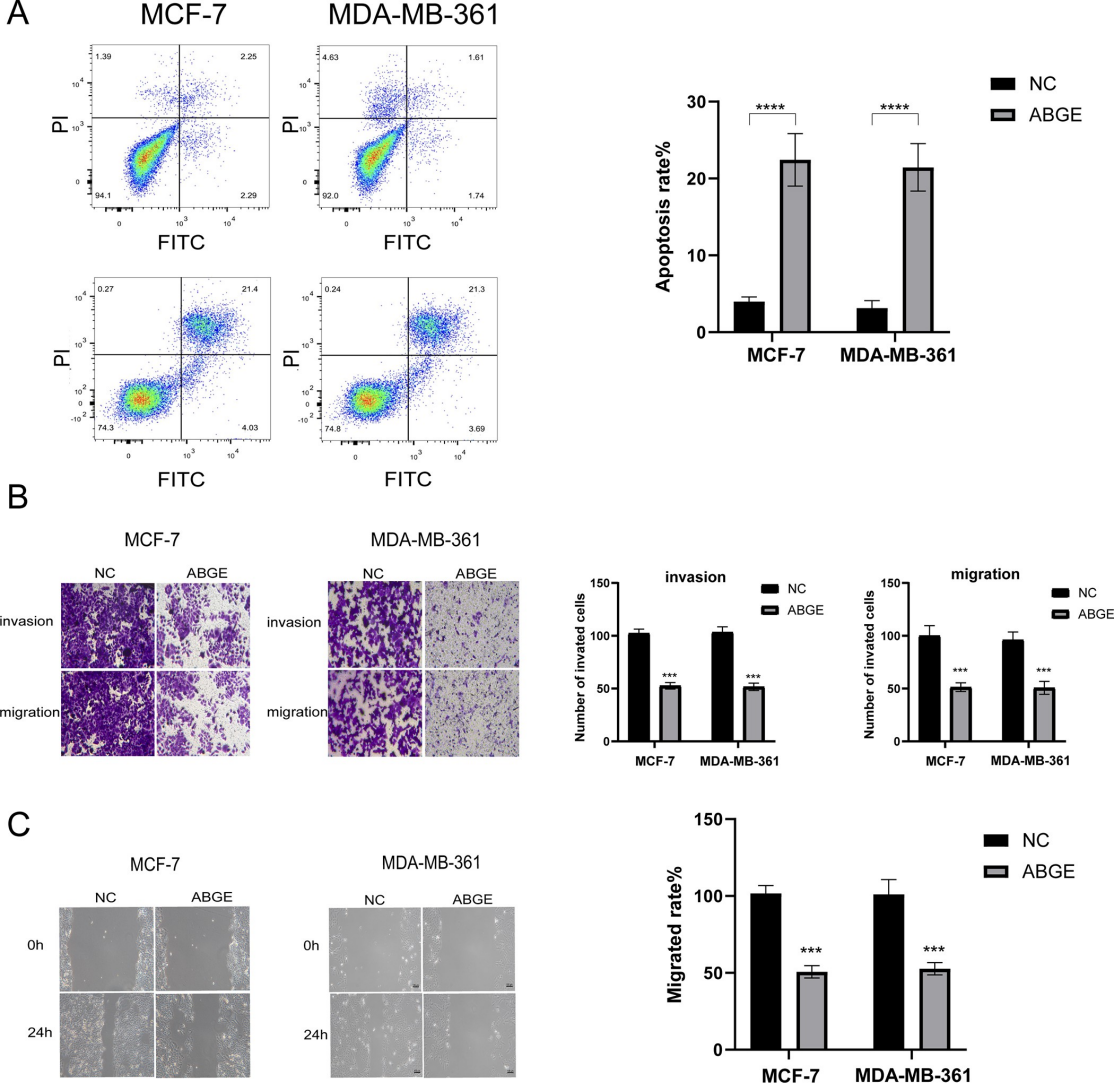
ABGE promoted apoptosis of breast cells. ABGE inhibited invasion, and migration of breast cells. (a)Flow cytometry analysis was used to assess apoptotic rate of MCF-7 and MDA-MB-361 cells. (b),The invasion ability of breast cancer cells was evaluated using Transwell assay. (magnification 200×). (c),Representative images and relative quantification of cell migration, as measured using wound healing assay. (magnification 100×). *** indicates *p* value less than 0.01.

To further investigate the roles of ABGE on cell apoptosis, migration, and invasion, we performed flow cytometry cell apoptosis assay, wound healing assay, and Transwell migration and invasion assay. The results showed that the cell apoptosis rate of MCF-7 and MDA-MB-361 cells was significantly increased after 48h ABGE treatment (Fig 2A) (p<0.01). For cell migration, the results of Transwell migration and wound healing assay showed that the migratory ability of MCF-7 and MDA-MB-361 cells treated with ABGE was significantly decreased. The assay suggested ABGE impaired the invasive ability of ER+ breast cancer cells (Fig 2B and 2C).

### ABGE inhibited epithelial-mesenchymal transition in ER+ breast cancer cells

**Fig 3:**
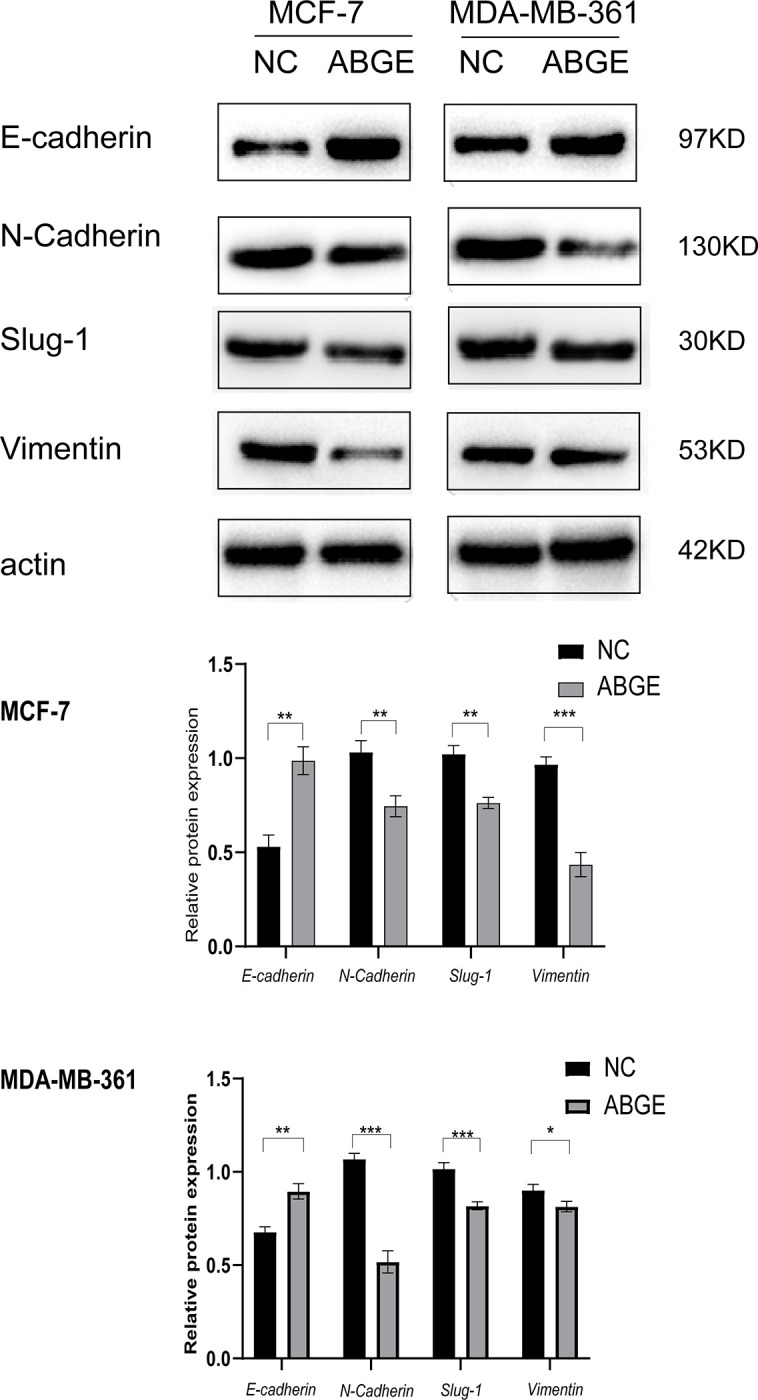
MCF-7 and MDA-MB-361 cells were cultured with ABGE-containing medium for 24 h. Western blot was performed to test the levels of EMT related markers. *indicates *p* value less than 0.05, **/*** indicates *p* value less than 0.01.

EMT is a necessary process for the metastasis of breast cancer cells. As ABGE inhibited the metastasis of ER+ breast cancer cells, we next explored the effect of ABGE on the EMT process. Several critical proteins involved in the EMT process were detected by Western blot analysis. As shown in Fig 3, after ABGE treatment for 24h, the protein expression of E-cadherin was dramatically upregulated, while the expression of N-cadherin, Slug-1, and Vimentin was significantly downregulated. These results indicated that ABGE hindered the EMT process in MCF-7 and MDA-MB-361 cells.

### ABGE decreases the expression of anti-apoptotic proteins MCL-1 and BCL-2 and increases the expression of pro-apoptotic proteins BIM and BAK in ER+ breast cancer cells

**Fig 4:**
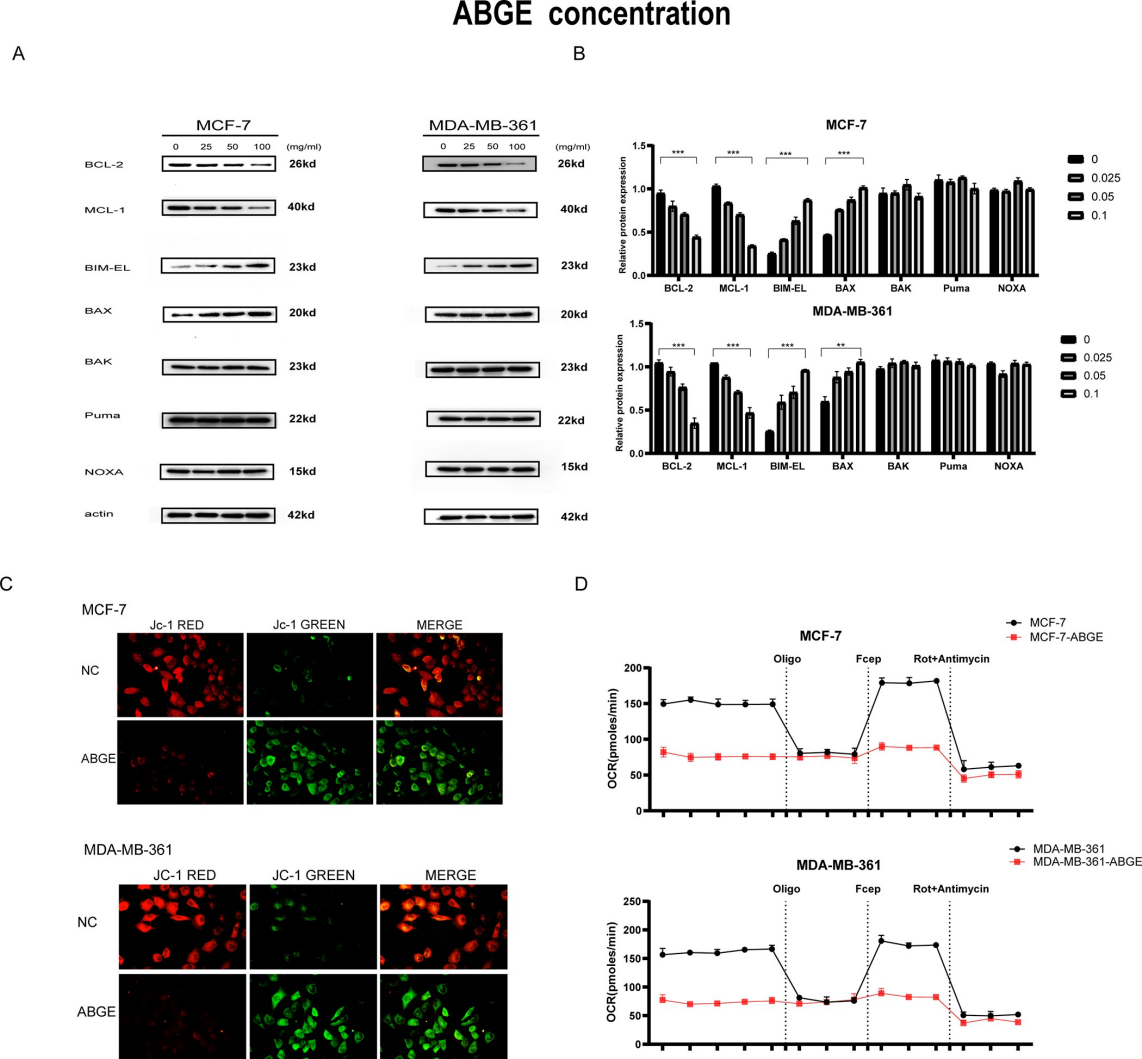
ABGE inhibits MCL-1 expression. (a),MCF-7 cells were treated with 25, 50 and 100mg/ml ABGE for 4h. (b),MDA-MB-361 cells were treated with 25, 50 and 100mg/ml ABGE for 4h. Bcl-2 family proteins were detected by western blot. (c)(d),mitochondrial membrane potential detected by JC-1 fluorescent staining and performed oxygen consumption assays. Mcl-1,BIM-EL,Puma and NOXA proteins were evaluated by western blot. **/*** indicates *p* value less than 0.01.

Members of the BCL-2 family are responsible for the fate of cell apoptosis. Since ABGE promoted apoptosis, we further explored the expression of BCL-2 family members before and after ABGE treatment. Fig 4 showed that after 4h 0.025, 0.05, and 100mg/ml ABGE treatment, the expression of anti-apoptotic protein MCL-1 and BCL-2 was decreased compared to the control group. Besides, the expression of pro-apoptotic protein BIM and the apoptosis executioner Bax was dramatically increased, and Bak, PUMA, and NOXA expression were not altered, suggesting that the changes of these proteins are partially the reason why ABGE promotes apoptosis in ER+ breast cancer cells. Next, to explore whether ABGE impairs mitochondrial function in ER+ breast cells, we detected mitochondrial membrane potential using JC-1 fluorescent staining and performed oxygen consumption assays. As shown in Fig 4C, compared with the control group, the red fluorescence was significantly weakened and the green fluorescence was significantly enhanced in the ABGE group, suggesting that ABGE impaired the mitochondrial function of MCF-7 and MDA-MB-361 cells. The results of the OCR assay were presented in Fig 4D. The results indicated that mitochondrial OCR was reduced at basal and after FCCP treatment in the ABGE group, suggesting ABGE impaired mitochondrial respiratory function.

### ABGE decreases MCL-1 expression through ROS-induced JNK activation

**Fig 5:**
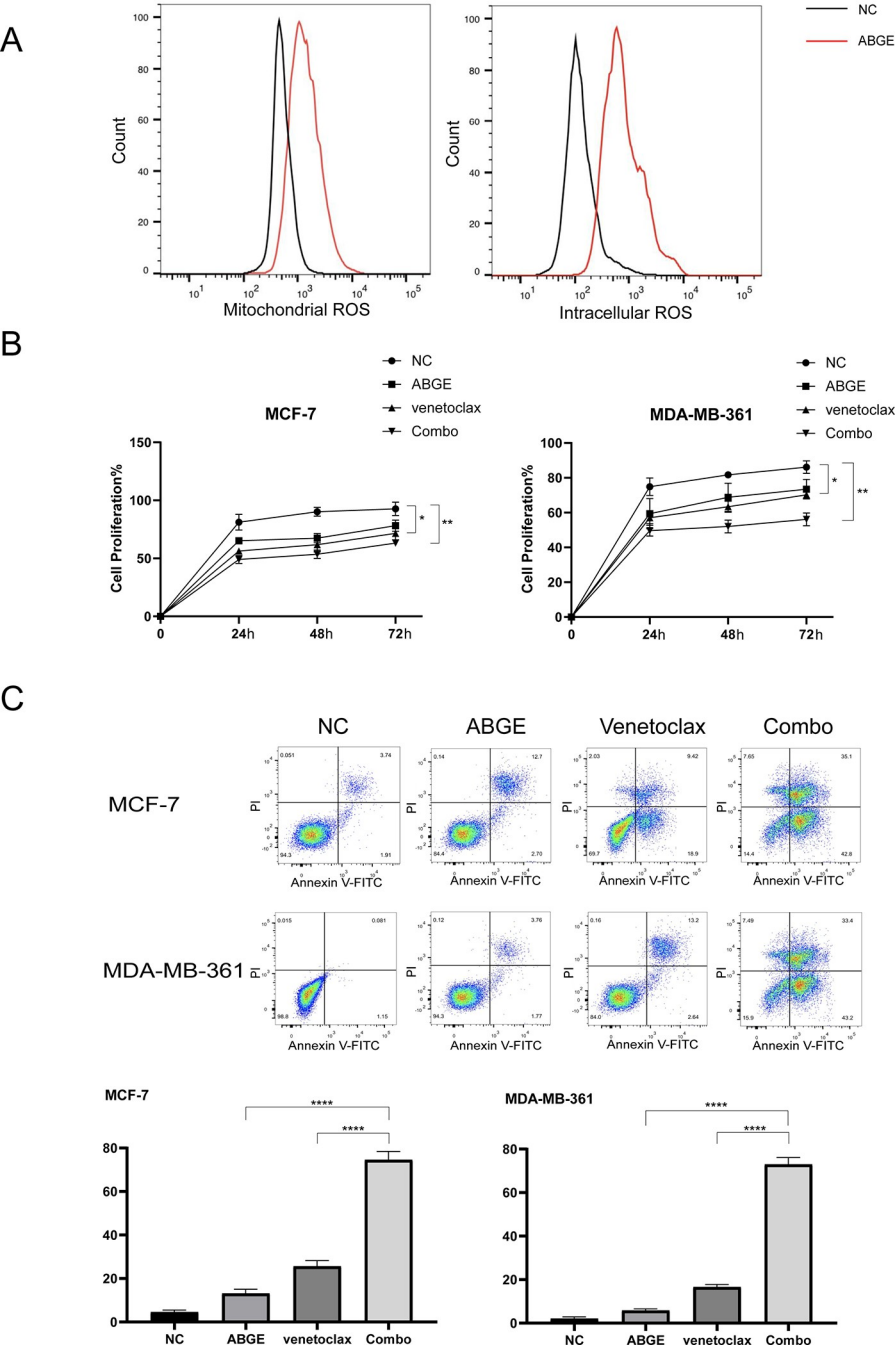
ABGE induces Mcl-1 degradation. ABGE +venetoclax induced significantly higher levels of apoptosis than ABGE or venetoclax alone in MCF-7 and MDA-MB-361 cells. (a),Mitochondrial ROS and Intracellular ROS levels were detected by flow cytometry. (b),Cells proliferation were detected by MTT Assay. (c),The combination of ABGE and venetoclax led to the greatest percentage of Annexin-V-FITC-positive cells, and the percentage was significantly different from that of NC, ABGE-treated and venetoclax-treated cells for both cell lines. *indicates *p* value less than 0.05, **/**** indicates *p* value less than 0.01.

**Fig 6:**
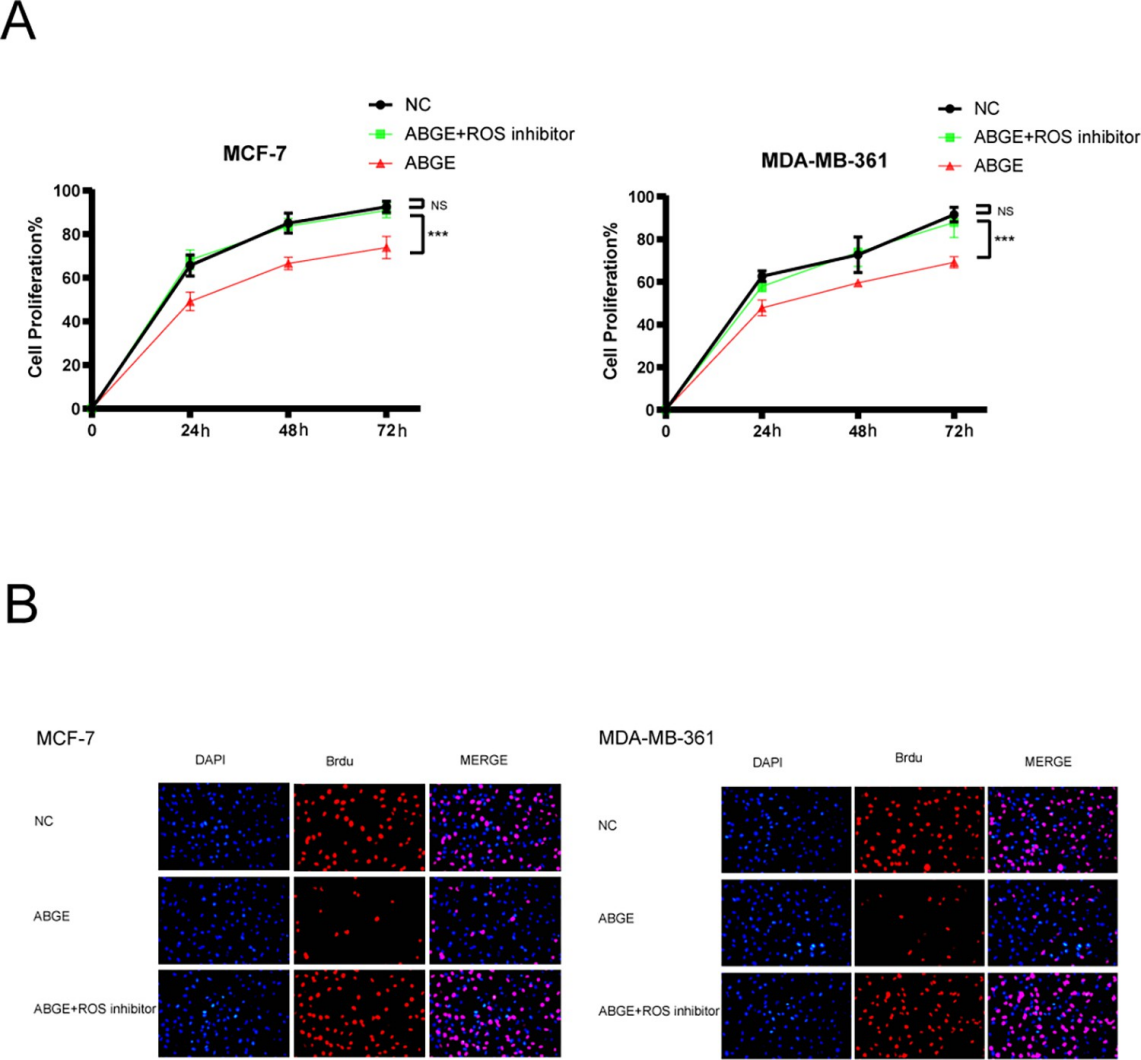
The proliferation inhibitory effect of ABGE on MCF-7 and MDA-MB-361. (a),Cells proliferation were detected by MTT Assay. (b),The proliferation of MCF-7 and MDA-MB-361 cells was examined by BrdU assay and CFSE assay, respectively. *** indicates *p* value less than 0.01.

Previous studies have shown that decreased expression of MCL-1 and increased cell apoptosis were associated with increased intracellular ROS. To explore whether the decreased expression of MCL-1 and increased cell apoptosis after ABGE treatment were related to the ROS in ER+ breast cancer, we examined the mitochondrial and intracellular ROS levels in MCF-7 cells. The results were shown in Fig 5A. After 24h ABGE treatment, both mitochondrial and intracellular ROS increased significantly compared to the control groups, suggesting a role of ROS in ABGE-induced ER+ breast cancer cell death. To further confirm that ABGE exerted its antitumor effect by increasing ROS, the ROS inhibitor N-acetyl-L-cysteine (NAC) was used. As shown in Fig 6A and 6B, the proliferation inhibitory effect of ABGE on MCF-7 and MDA-MB-361 almost disappeared after 30 min of NAC pretreatment. These results suggested that ABGE exerts its antitumor effect in ER+ breast cancer cells by increasing cellular ROS (Fig 5B and 5C).

**Fig 7:**
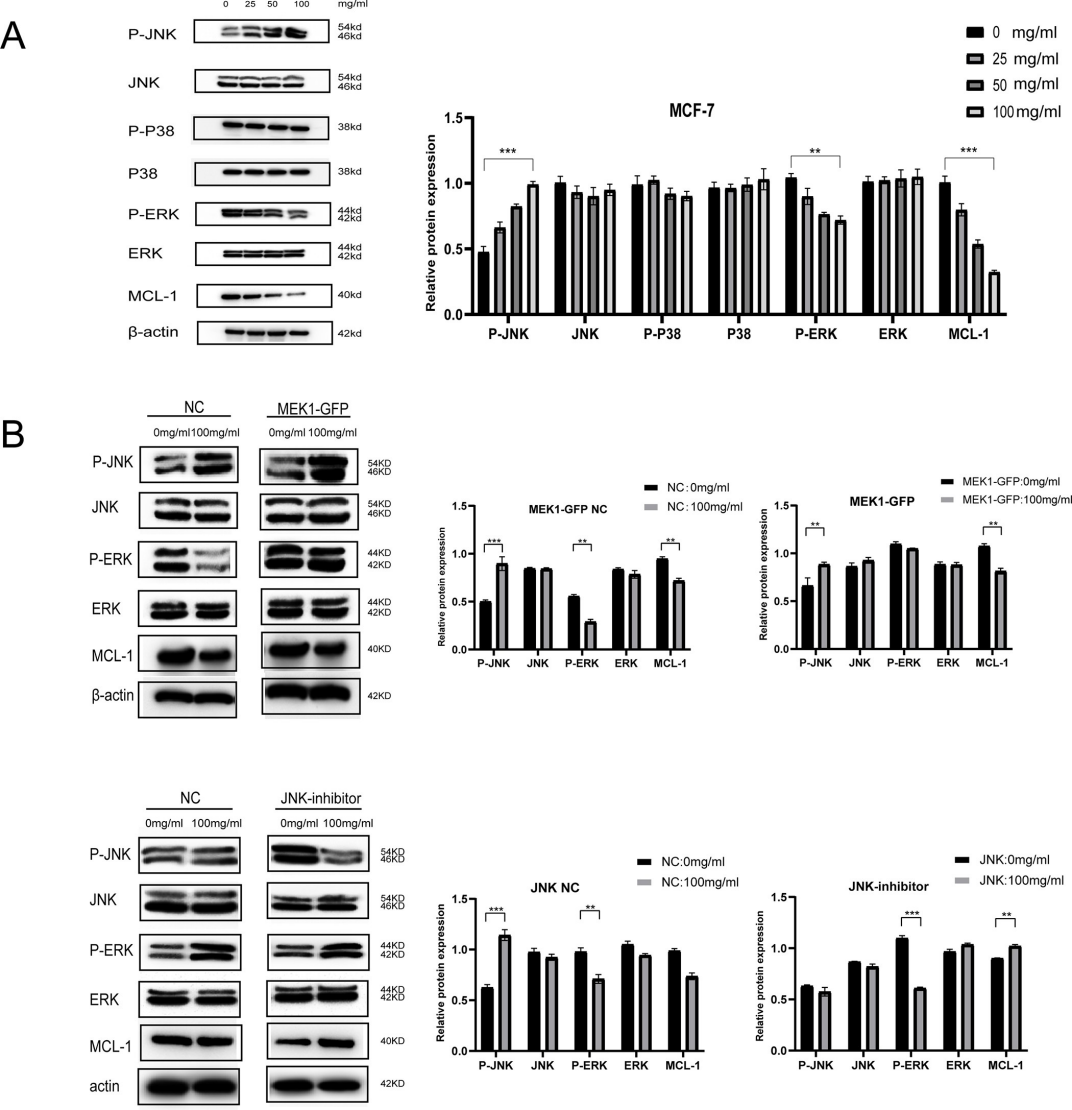
The expression of several essential proteins related to signaling pathways that are downstream of ROS generation. (a),MCF-7 cells were treated with indicated concentrations of 25, 50 and 100mg/ml ABGE for 24 h. The levels of P-JNK, JNK, P-P38, P38, P-ERK, ERK, Mcl-1 and β-actin were determined by western blot analysis. (b),Overexpression of MEK1-GFP and inhibition of JNK signalling in MCF-7 cells. P-JNK, JNK, P-ERK, ERK and MCL-1 were determined by western blot analysis. **/*** indicates *p* value less than 0.01.

We further explored the expression of several essential proteins related to signaling pathways that are downstream of ROS generation. We found that the expression of p-JNK was significantly upregulated, while the expression of p-ERK and MCL-1 was significantly downregulated. The expression of p38, p-p38, JNK, and ERK were not significantly changed (Fig 7A). According to these results, we hypothesized that JNK and ERK/MAPK signaling pathways might account for ROS-mediated MCL-1 downregulation and apoptosis promotion in ER+ breast cancer cells.

We overexpressed MEK1-GFP and inhibited JNK signaling in MCF-7 cells to validate our hypothesis. Fig 7B showed that ERK activation could not rescue the MCL-1 downregulation caused by ABGE treatment. At the same time, JNK inhibition successfully prevented ABGE-induced MCL-1 reduction, indicating ABGE-induced MCL-1 downregulation was regulated through the ROS-JNK pathway.

### ABGE enhances the anti-tumor effects of BCL-2 inhibitor venetoclax in ER+ breast cancer cells

The clinical trials of venetoclax show that it synergizes with hormonal therapy in ER+ breast cancer models. Previous studies have demonstrated that the high expression of MCL-1 is an important reason for the insensitivity of tumor cells to venetoclax. The present study showed that ABGE effectively reduced the MCL-1 expression. Therefore, we sought to investigate whether ABGE synergizes with venetoclax to eliminate ER+ breast cancer cells. The MTT assay and the flow cytometry apoptosis assay were carried out in MCF-7 and MDA-MB-361 cells treated with ABGE or venetoclax or ABGE+venetoclax (Combo) for 24,48 and 72h. The results showed that the proliferation of cells in the combination group was significantly inhibited (Fig 7A), while apoptosis was promoted (Fig 7B). The results suggested that ABGE synergized with venetoclax in killing ER+ breast cancer cells.

## Discussion

Most breast cancers are ER+ positive [9]. The treatment of ER+ breast cancer involves suppressing estrogen production and/or targeting ER directly (endocrine therapy). Despite substantial reductions in breast cancer mortality and recurrence due to endocrine therapy, resistance to this treatment persists. The mechanisms leading to endocrine resistance of ER+ breast cancer mainly include the continuous activation of some cancer-promoting pathways, such as MAPK [10] and PI3K [11] signaling pathways, somatic mutations [12], epigenetic [13] and metabolic changes [14], etc. Although neoadjuvant chemotherapy, radiotherapy, and immunotherapy have improved the prognosis of ER+ breast cancer patients resistant to endocrine therapy, the subsequent occurrence of drug resistance and side effects still make these treatments less effective. As a result, it is imperative to find more effective treatment strategies for breast cancer with fewer side effects.

Natural dietary products are effective in preventing and treating cancer [15, 16]. One such ingredient is aged black garlic, which contains many bioactive substances such as SAC and SAMC. In previous studies, it has been proven to have a variety of functions including anti-inflammatory [17], antioxidant [18], and anti-tumor [2–5, 19]. In gastric carcinoma, ABGE inhibits tumor growth, invasion, and metastasis [2]. In U937 leukemia cells, ABGE induces cell apoptosis in a caspase-dependent manner [3]. In colon cancer, ABGE impaired cell growth by inhibiting PI3K/AKT pathway [5]. Although a previous study showed that ABGE inhibits the proliferation of MCF-7 [4], the mechanisms underlying this phenomenon remain unclear.

In the present study, we observed that ABGE inhibited the proliferation of ER+ breast cancer cells, promoted apoptosis, and inhibited invasion and migration. Mechanically, ABGE inhibited the EMT process, promoted the expression of pro-apoptotic protein BIM and apoptotic executioner BAX, and decreased the expression of anti-apoptotic proteins BCL-2 and MCL-1. In addition, further exploration revealed that ABGE reduced MCL-1 expression through the ROS-JNK signaling pathway.

Previous studies have found that Annurca Apple polyphenol Extract [20], Juglanin [21] and Curcumin Derivative WZ35ROS [22] inhibited the growth of breast cancer cells by activating the ROS/JNK signaling pathway. MCL-1 degradation induced by ROS/JNK signaling activation has been reported in the lung [23] and liver cancer [24]. Amino acids Thr163 and Ser121 of MCL-1 were phosphorylated by JNK, causing MCL-1 to be inactivated and degraded [8]. In the present study, we proposed for the first time that the degradation of MCL-1 by ABGE was achieved through ROS-dependent activation of JNK signaling.

Combining chemotherapy agents plays a vital role in treating ER+ breast cancer. Combination therapeutic strategies synergistically kill tumor cells and reduce the adverse effects caused by excessive doses of a single agent. Previous studies have shown that low-dose dexamethasone combined with venetoclax and tamoxifen in metastatic ER+ breast cancer significantly increased life expectancy compared with venetoclax or tamoxifen administration alone [7]. In the present study, we found that co-administration of ABGE and venetoclax had synergistic effects in killing ER+ breast cancer cells. The combination index was far less than 1, indicating the combination exhibited an excellent tumor-killing effect.

Since ABGE is extracted from natural dietary products with few side effects, our study provides a new therapeutic option for treating ER+ breast cancer.

## Supporting information

S1 Fig Normal mammary cell line MCF-10A was used to detect whether ABGE is toxic to normal breast cells. And we did not observe an inhibitory effect of ABGE on their growth.
(TIF)

S1 File (PDF)

## References

[1] Kimura S, Tung Y-C, Pan M-H, Su N-W, Lai Y-J, et al. Black garlic: A critical review of its production, bioactivity, and application. Journal of Food and Drug Analysis. 2017;25(1):62–70. doi: 10.1016/j.jfda.2016.11.003 28911544 PMC9333422

[2] Wang X, Jiao F, Wang QW, Wang J, Yang K, Hu RR, et al. Aged black garlic extract induces inhibition of gastric cancer cell growth in vitro and in vivo. Mol Med Rep. 2012;5(1):66–72. Epub 2011/09/17. doi: 10.3892/mmr.2011.588 . 21922142

[3] Park C, Park S, Chung YH, Kim GY, Choi YW, Kim BW, et al. Induction of apoptosis by a hexane extract of aged black garlic in the human leukemic U937 cells. Nutr Res Pract. 2014;8(2):132–7. Epub 2014/04/18. doi: 10.4162/nrp.2014.8.2.132 ; PubMed Central PMCID: PMC3988500. 24741395 PMC3988500

[4] Purev U, Chung MJ, Oh DH. Individual differences on immunostimulatory activity of raw and black garlic extract in human primary immune cells. Immunopharmacol Immunotoxicol. 2012;34(4):651–60. Epub 2012/01/21. doi: 10.3109/08923973.2011.649288 . 22260639

[5] Dong M, Yang G, Liu H, Liu X, Lin S, Sun D, et al. Aged black garlic extract inhibits HT29 colon cancer cell growth via the PI3K/Akt signaling pathway. Biomed Rep. 2014;2(2):250–4. Epub 2014/03/22. doi: 10.3892/br.2014.226 ; PubMed Central PMCID: PMC3917757. 24649105 PMC3917757

[6] Dawson SJ, Makretsov N, Blows FM, Driver KE, Provenzano E, Le Quesne J, et al. BCL2 in breast cancer: a favourable prognostic marker across molecular subtypes and independent of adjuvant therapy received. Br J Cancer. 2010;103(5):668–75. Epub 2010/07/29. doi: 10.1038/sj.bjc.6605736 ; PubMed Central PMCID: PMC2938244. 20664598 PMC2938244

[7] Lok SW, Whittle JR, Vaillant F, I CE, Lo LL, Policheni AN, et al. A Phase Ib Dose-Escalation and Expansion Study of the BCL2 Inhibitor Venetoclax Combined with Tamoxifen in ER and BCL2-Positive Metastatic Breast Cancer. Cancer Discov. 2019;9(3):354–69. Epub 2018/12/07. doi: 10.1158/2159-8290.CD-18-1151 . 30518523

[8] Inoshita S, Takeda K, Hatai T, Terada Y, Sano M, Hata J, et al. Phosphorylation and inactivation of myeloid cell leukemia 1 by JNK in response to oxidative stress. Journal of Biological Chemistry. 2002;277(46):43730–4. doi: 10.1074/jbc.M207951200 12223490

[9] Hanker AB, Sudhan DR, Arteaga CL. Overcoming Endocrine Resistance in Breast Cancer. Cancer Cell. 2020;37(4):496–513. Epub 2020/04/15. doi: 10.1016/j.ccell.2020.03.009 ; PubMed Central PMCID: PMC7169993. 32289273 PMC7169993

[10] Razavi P, Chang MT, Xu G, Bandlamudi C, Ross DS, Vasan N, et al. The Genomic Landscape of Endocrine-Resistant Advanced Breast Cancers. Cancer Cell. 2018;34(3):427–38.e6. Epub 2018/09/12. doi: 10.1016/j.ccell.2018.08.008 ; PubMed Central PMCID: PMC6327853. 30205045 PMC6327853

[11] Miller TW, Hennessy BT, González-Angulo AM, Fox EM, Mills GB, Chen H, et al. Hyperactivation of phosphatidylinositol-3 kinase promotes escape from hormone dependence in estrogen receptor-positive human breast cancer. J Clin Invest. 2010;120(7):2406–13. Epub 2010/06/10. doi: 10.1172/JCI41680 ; PubMed Central PMCID: PMC2898598. 20530877 PMC2898598

[12] Angus L, Smid M, Wilting SM, van Riet J, Van Hoeck A, Nguyen L, et al. The genomic landscape of metastatic breast cancer highlights changes in mutation and signature frequencies. Nat Genet. 2019;51(10):1450–8. Epub 2019/10/02. doi: 10.1038/s41588-019-0507-7 ; PubMed Central PMCID: PMC6858873. 31570896 PMC6858873

[13] Dagogo-Jack I, Shaw AT. Tumour heterogeneity and resistance to cancer therapies. Nat Rev Clin Oncol. 2018;15(2):81–94. Epub 2017/11/09. doi: 10.1038/nrclinonc.2017.166 . 29115304

[14] Zinger L, Merenbakh-Lamin K, Klein A, Elazar A, Journo S, Boldes T, et al. Ligand-binding Domain-activating Mutations of ESR1 Rewire Cellular Metabolism of Breast Cancer Cells. Clin Cancer Res. 2019;25(9):2900–14. Epub 2019/02/09. doi: 10.1158/1078-0432.CCR-18-1505 . 30733228

[15] Zhou Y, Li Y, Zhou T, Zheng J, Li S, Li HB. Dietary Natural Products for Prevention and Treatment of Liver Cancer. Nutrients. 2016;8(3):156. Epub 2016/03/16. doi: 10.3390/nu8030156 ; PubMed Central PMCID: PMC4808884. 26978396 PMC4808884

[16] Zheng J, Zhou Y, Li Y, Xu DP, Li S, Li HB. Spices for Prevention and Treatment of Cancers. Nutrients. 2016;8(8). Epub 2016/08/17. doi: 10.3390/nu8080495 ; PubMed Central PMCID: PMC4997408. 27529277 PMC4997408

[17] Jeong YY, Ryu JH, Shin JH, Kang MJ, Kang JR, Han J, et al. Comparison of Anti-Oxidant and Anti-Inflammatory Effects between Fresh and Aged Black Garlic Extracts. Molecules. 2016;21(4):430. Epub 2016/04/05. doi: 10.3390/molecules21040430 ; PubMed Central PMCID: PMC6274159. 27043510 PMC6274159

[18] Sato E, Kohno M, Hamano H, Niwano Y. Increased anti-oxidative potency of garlic by spontaneous short-term fermentation. Plant Foods Hum Nutr. 2006;61(4):157–60. Epub 2006/11/01. doi: 10.1007/s11130-006-0017-5 . 17075725

[19] Shin D-Y, Yoon M-K, Choi Y-W, Gweon O-C, Kim J-I, Choi T-H, et al. Effects of aged black garlic extracts on the tight junction permeability and cell invasion in human gastric cancer cells. Journal of Life Science. 2010;20(4):528–34.

[20] Vuoso DC, D’Angelo S, Ferraro R, Caserta S, Guido S, Cammarota M, et al. Annurca apple polyphenol extract promotes mesenchymal-to-epithelial transition and inhibits migration in triple-negative breast cancer cells through ROS/JNK signaling. Sci Rep. 2020;10(1):15921. Epub 2020/09/29. doi: 10.1038/s41598-020-73092-2 ; PubMed Central PMCID: PMC7522716. 32985606 PMC7522716

[21] Sun ZL, Dong JL, Wu J. Juglanin induces apoptosis and autophagy in human breast cancer progression via ROS/JNK promotion. Biomed Pharmacother. 2017;85:303–12. Epub 2016/12/03. doi: 10.1016/j.biopha.2016.11.030 . 27899257

[22] Wang L, Wang C, Tao Z, Zhao L, Zhu Z, Wu W, et al. Curcumin derivative WZ35 inhibits tumor cell growth via ROS-YAP-JNK si gnaling pathway in breast cancer. J Exp Clin Cancer Res. 38(1):460. doi: 10.1186/s13046-019-1424-4 PubMed Central PMCID: PMC6842168. 31703744 PMC6842168

[23] Chiu WH, Luo SJ, Chen CL, Cheng JH, Hsieh CY, Wang CY, et al. Vinca alkaloids cause aberrant ROS-mediated JNK activation, Mcl-1 downregulation, DNA damage, mitochondrial dysfunction, and apoptosis in lung adenocarcinoma cells. Biochem Pharmacol. 2012;83(9):1159–71. Epub 2012/01/31. doi: 10.1016/j.bcp.2012.01.016 . 22285910

[24] Sun L, Jiang Y, Yan X, Dai X, Huang C, Chen L, et al. Dichloroacetate enhances the anti-tumor effect of sorafenib via modulating the ROS-JNK-Mcl-1 pathway in liver cancer cells. Exp Cell Res. 2021;406(1):112755. Epub 2021/08/02. doi: 10.1016/j.yexcr.2021.112755 . 34332981

