# Supplementary figures and images for: Aged black garlic extract inhibits the growth of estrogen receptor-positive breast cancer cells by downregulating MCL-1 expression through the ROS-JNK pathway
Source: PLoS One. 2023 Jun 23;18(6):e0286454. doi: 10.1371/journal.pone.0286454 (PMC10289325; doi:10.1371/journal.pone.0286454)

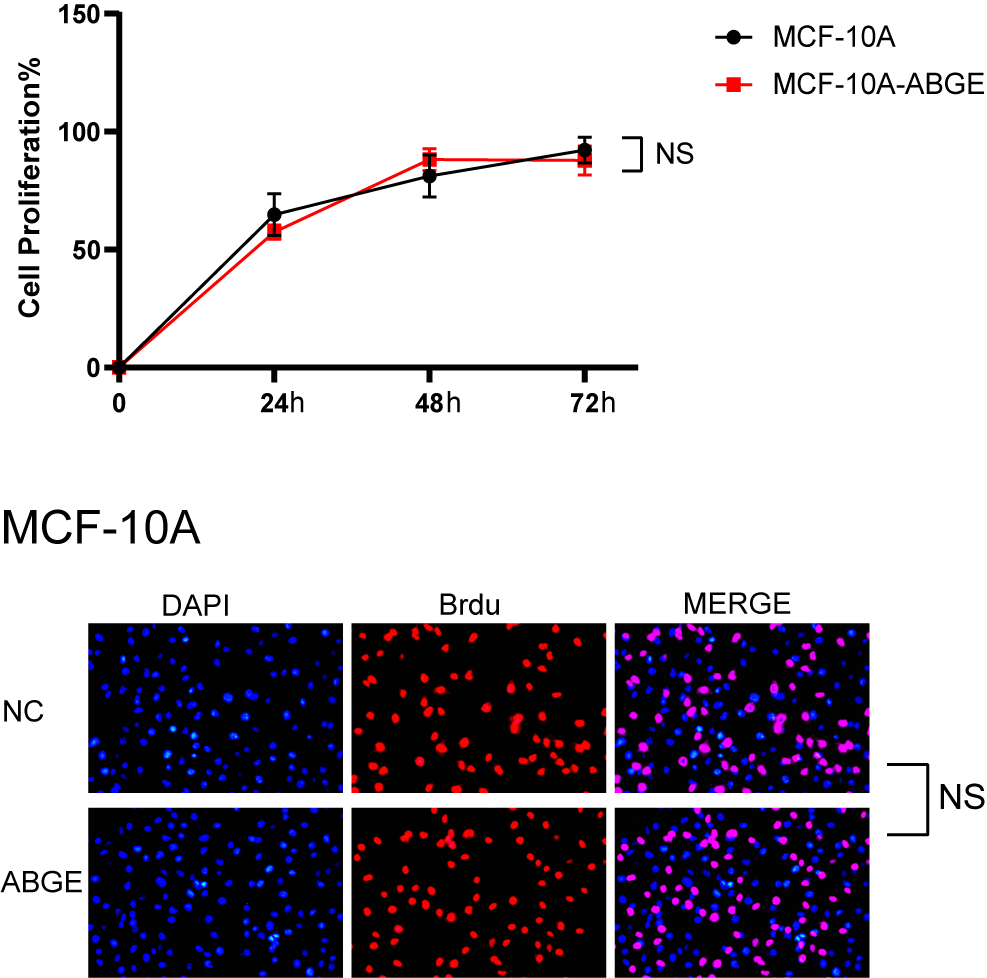

Supplement: S1 Fig — And we did not observe an inhibitory effect of ABGE on their growth. (TIF) [file pone.0286454.s001.tif]
