# Supplementary material for: Aged black garlic extract inhibits the growth of estrogen receptor-positive breast cancer cells by downregulating MCL-1 expression through the ROS-JNK pathway
Source: PLoS One. 2023 Jun 23;18(6):e0286454. doi: 10.1371/journal.pone.0286454 (PMC10289325; doi:10.1371/journal.pone.0286454)

fig3 Western-blot of EMT related markers proteins in MCF-7 and MDA-MB-361 cells.

actin

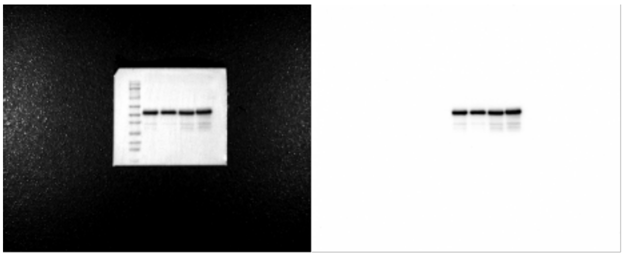

E-Cadherin

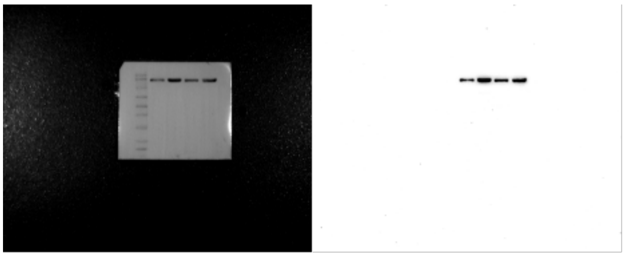

N-Cadherin

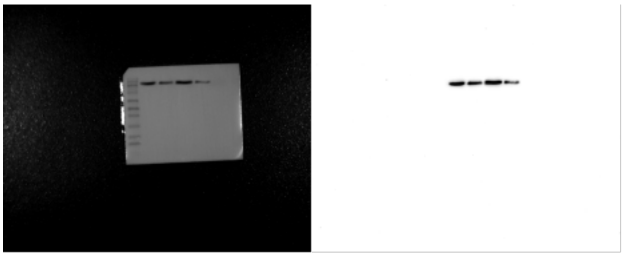

Slug-1

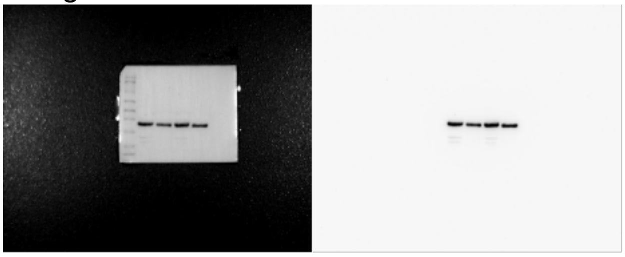

Vimentin

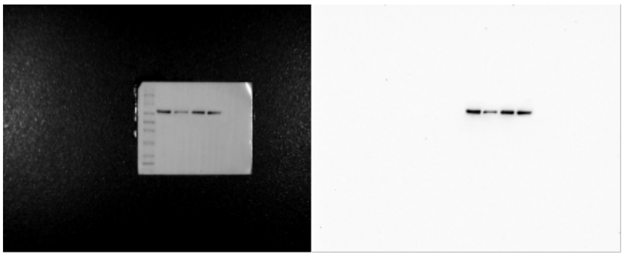

fig4A Western-blot of Bcl-2 family proteins in MCF-7 cells.

actin

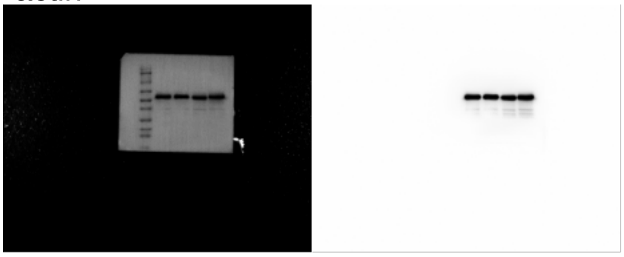

BAK

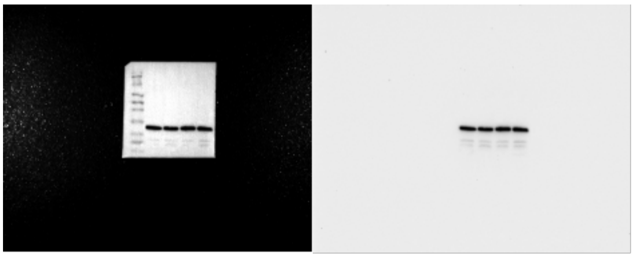

BAX

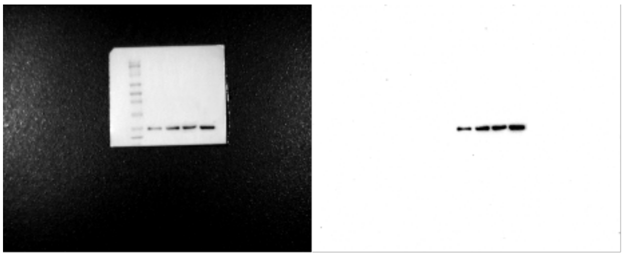

BCL-2

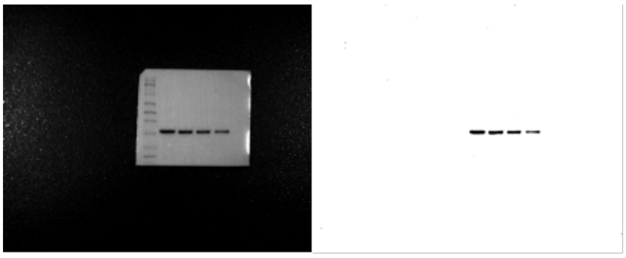

BIM-EL

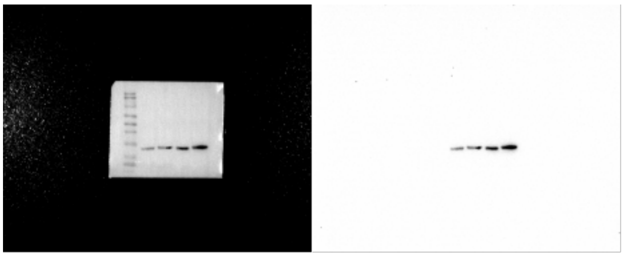

MCL-1

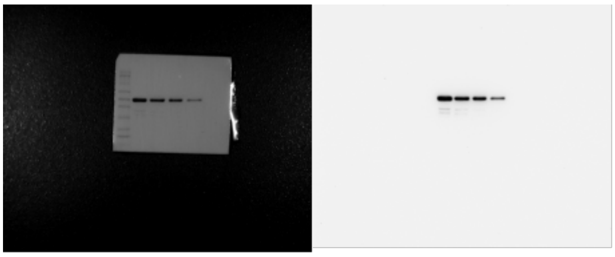

NOXA

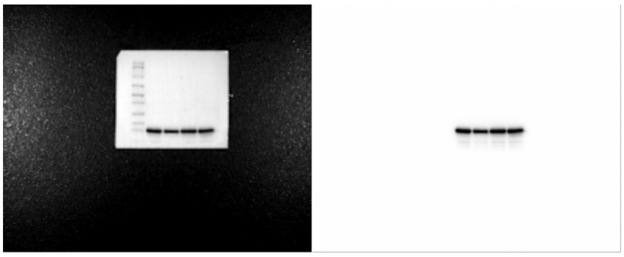

Puma

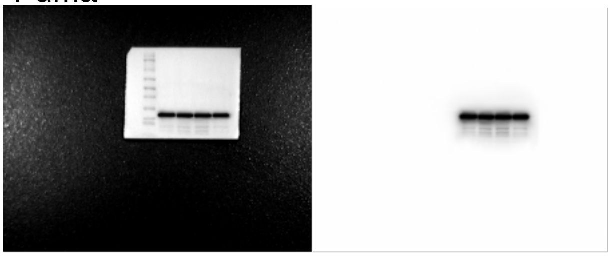

fig4B Western-blot of Bcl-2 family proteins in MDA-MB-361 cells.

actin

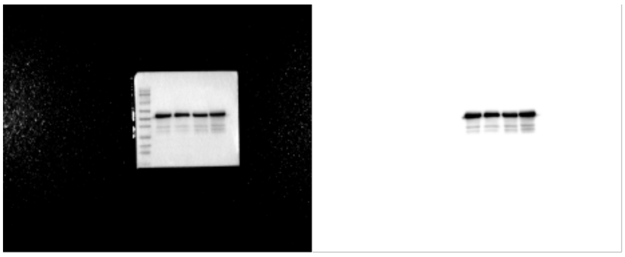

BAK

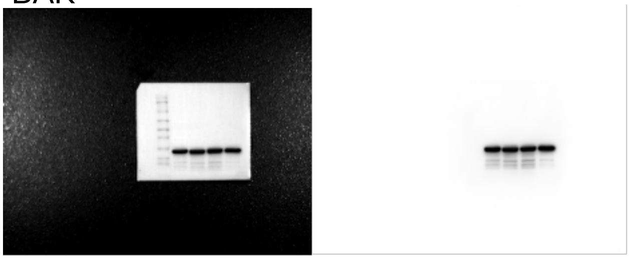

BAX

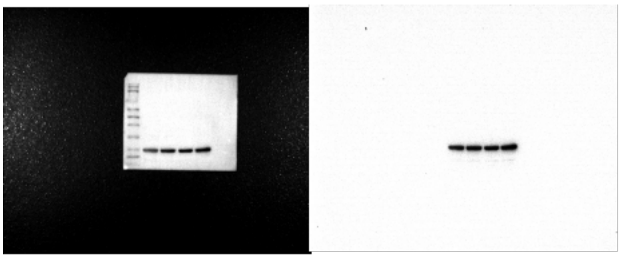

BCL-2

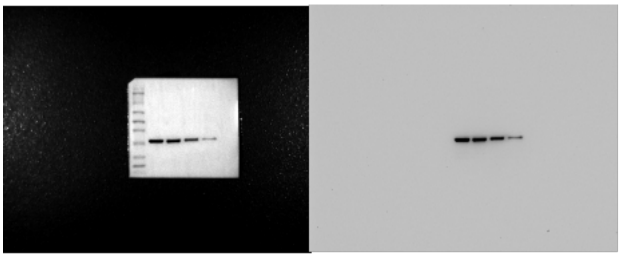

BIM-EL

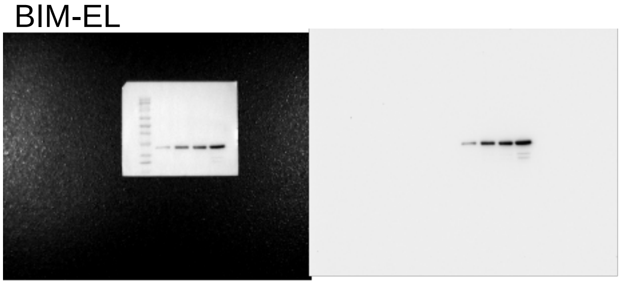

MCL-1

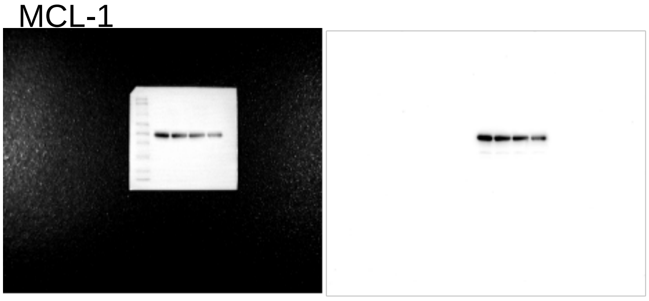

NOXA

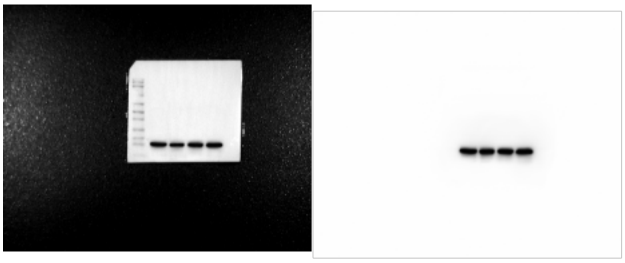

Puma

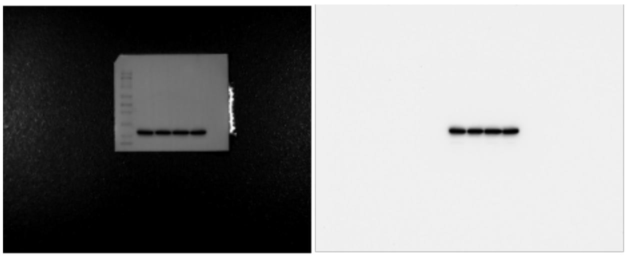

fig7A Western-blot of several essential proteins related to signaling pathways that are downstream of ROS generation in MCF-7 cells.

actin

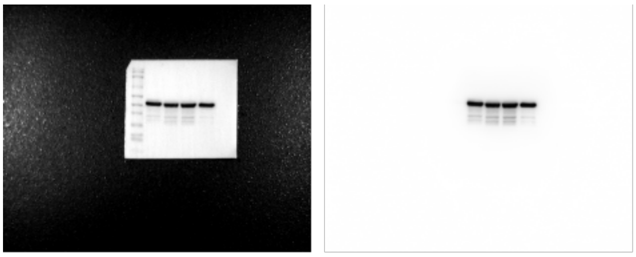

ERK

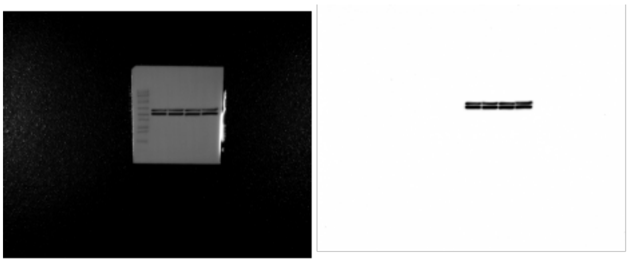

JNK

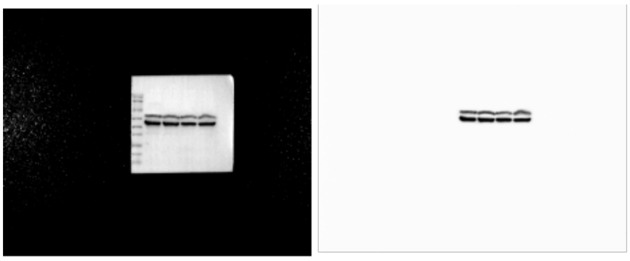

MCL-1

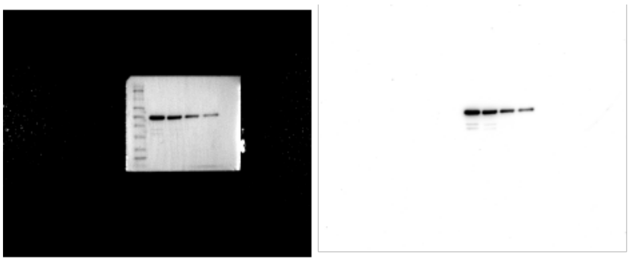

P38

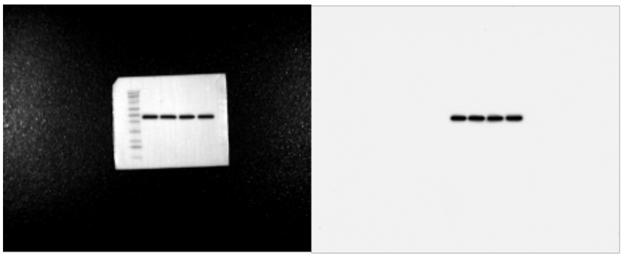

P-ERK

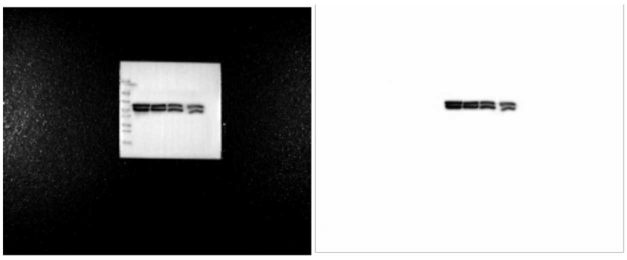

P-JNK

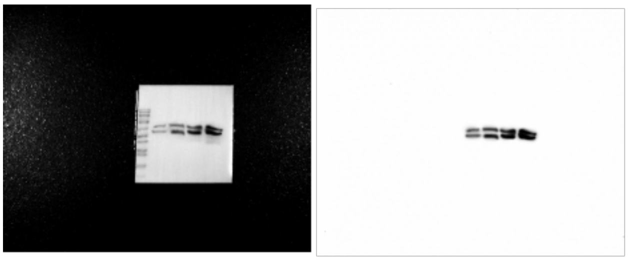

P-P38

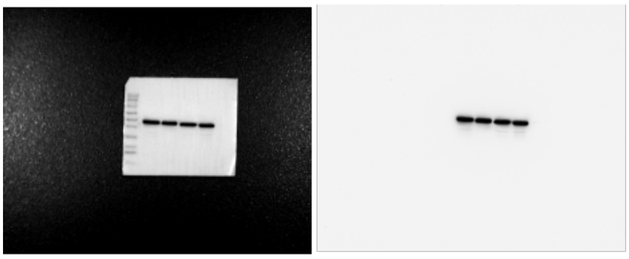

fig7B Western-blot of inhibited JNK signaling in MCF-7 cells

actin

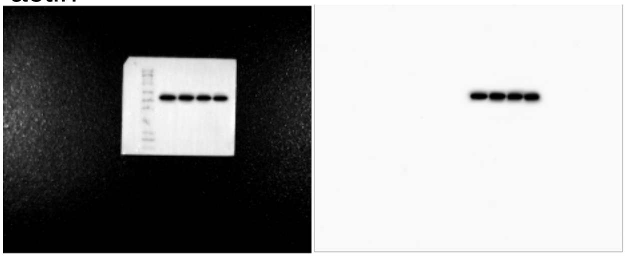

ERK

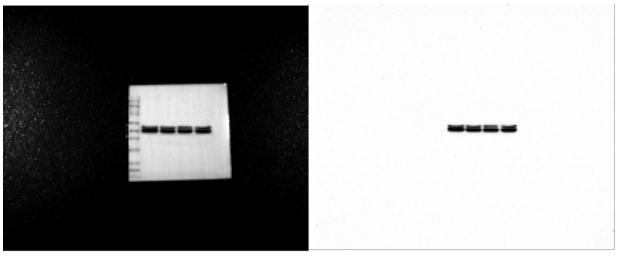

JNK

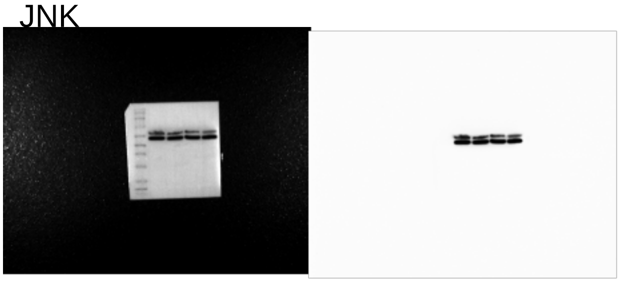

MCL-1

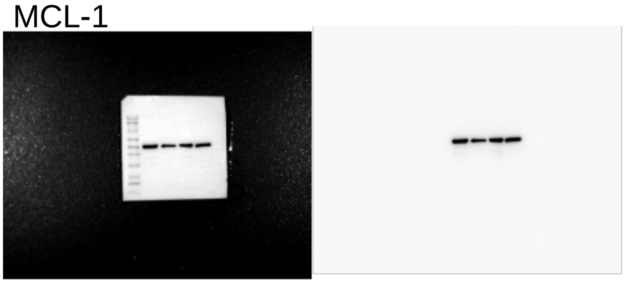

P-ERK

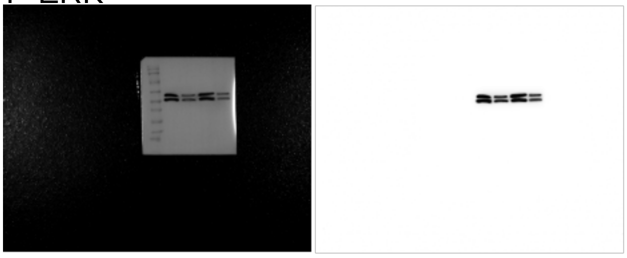

P-JNK

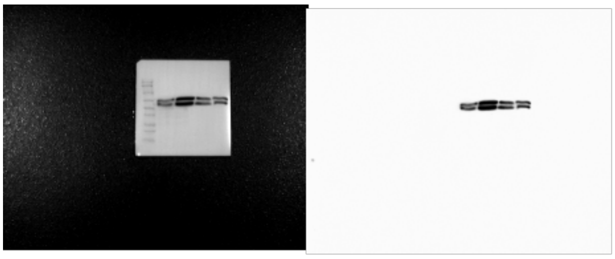

fig7B Western-blot of Overexpression of MEK1-GFP in MCF-7 cells.

actin

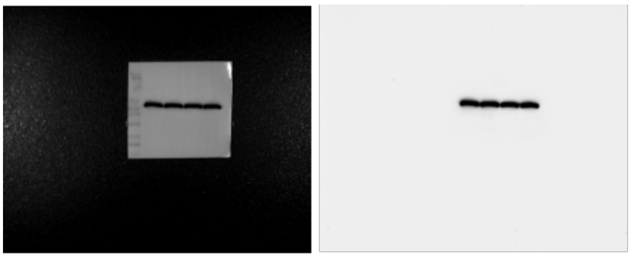

ERK

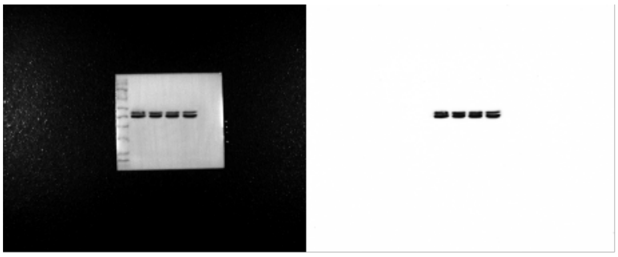

JNK

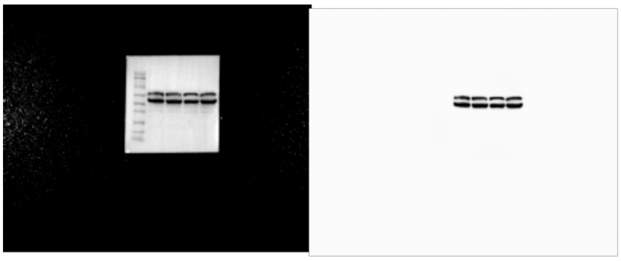

MCL-1

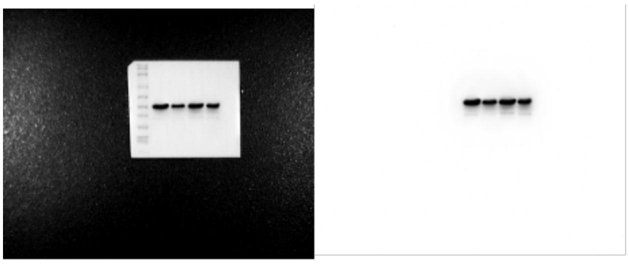

P-ERK

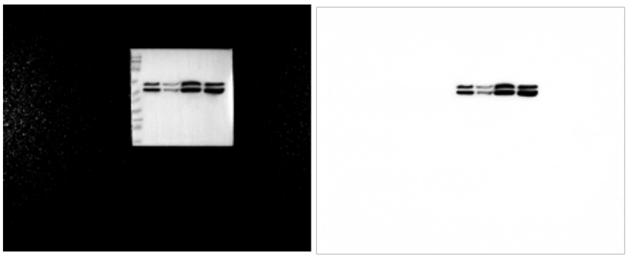

P-JNK

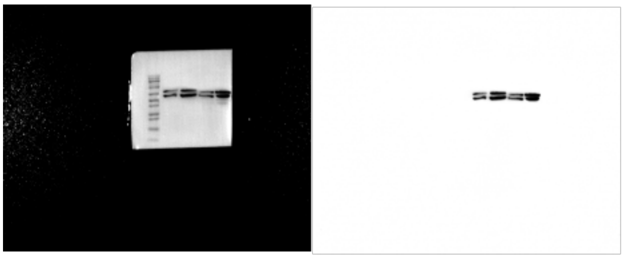

Supplement: S1 File — (PDF) [file pone.0286454.s002.pdf]
